# Prevalence of restless legs syndrome in migraine patients with and without aura: a cross-sectional, case-controlled study

**DOI:** 10.1186/s10194-016-0691-0

**Published:** 2016-10-21

**Authors:** Guan-Yu Lin, Yu-Kai Lin, Jiunn-Tay Lee, Meei-Shyuan Lee, Chun-Chieh Lin, Chia-Kuang Tsai, Chi-Hsin Ting, Fu-Chi Yang

**Affiliations:** 1 Department of Neurology, Tri-Service General Hospital, National Defense Medical Center, Taipei, Taiwan; 2 School of Public Health, National Defense Medical Center, Taipei, Taiwan; 3 Department of Internal Medicine, Taichung Armed Forces General Hospital, Taichung, Taiwan

**Keywords:** Migraine, Restless legs syndrome, Iron metabolism, Dopamine, Hospital anxiety and depression subscales, Pittsburgh sleep quality index

## Abstract

**Background:**

Although the comorbidity of migraine and restless legs syndrome (RLS) has been well-documented, the association between RLS and migraine frequency has yet to be elucidated. The present study aims to evaluate the prevalence of RLS among individuals who experience low-frequency, high-frequency, or chronic migraine presenting with and without aura.

**Methods:**

We conducted a cross-sectional, case-controlled study involving 505 participants receiving outpatient headache treatment. Standardized questionnaires were administered to collect information on experiences of migraine, RLS, sleep quality, anxiety, depression, and demographics. Participants were categorized into low-frequency (1–8/month), high-frequency (9–14/month), and chronic (≥15/month) headache groups. RLS was diagnosed according to the criteria outlined by the International RLS Study Group (IRLSSG). The Pittsburgh Sleep Quality Index (PSQI) and Hospital Anxiety and Depression Scale (HADS) were used to assess sleep quality and identify symptoms of anxiety and depression. Associations between migraine frequency and RLS prevalence were investigated using multivariate linear and logistic regression.

**Results:**

Univariate analysis revealed an effect of migraine frequency on RLS prevalence (*p* = 0.026), though this effect did not persist following adjustment for baseline characteristics (*p* = 0.256). The trend was robust in patients whose migraines presented with auras (*p*_univariate_ = 0.002; *p*_multivariate_ = 0.043) but not in those without auras (*p*_univariate_ and *p*_multivariate_ > 0.05). Higher anxiety [odds ratio (OR) = 1.18, *p* = 0.019] and sleep disturbance (OR = 1.17, *p* = 0.023) scores were associated with higher RLS prevalence.

**Conclusions:**

Higher migraine frequency correlates with a higher prevalence of RLS, particularly among patients with auras.

## Background

Migraine is a primary headache disorder that affects 10 ~ 20 % of the general population. The disorder is typically characterized by recurrent attacks of moderate-to-severe pulsating pain on one side of the head that may last anywhere from 4 to 72 hours. Migraine pain is often associated with photophobia, phonophobia, and/or nausea and vomiting, and may be further aggravated by physical activity [1, 2]. Research has revealed comorbidity of migraine and numerous other conditions, including cardiovascular disease, asthma, depression, stroke, epilepsy, and a number of other pain disorders [3–6]. In addition, migraineurs have been reported to have a higher than normal prevalence of sleep disorders, including the sleep-related movement disorder restless legs syndrome (RLS) [7, 8].

RLS, for which reported prevalence rates vary from 4 % to 29 % in the general population, is characterized by an unpleasant leg sensation that typically worsens at night and resolves with movement [9]. Approximately 70 % of RLS cases are primary idiopathic forms, perhaps related to some genetic predisposition, while the remaining cases are symptomatic of another primary condition, such as iron deficiency anemia, renal failure, depression, pregnancy, migraine, or even Parkinson’s disease [10]. Although the pathophysiology of RLS remains unclear [11, 12], current research indicates that dysfunction of the hypothalamic dopaminergic A11 nucleus may be involved [13]. Dopamine has also been implicated in migraine pathophysiology, especially with regard to premonitory symptoms such as yawning, food cravings, and gastrointestinal disturbances [14]. Interestingly, migraineurs with RLS more often experience premonitory symptoms than those without RLS, further supporting the notion of a dopaminergic link between migraine and RLS [15]. In addition, iron deposition in multiple deep brain nuclei has been addressed in migraineurs [16, 17], though not in those with RLS. Moreover, spreading reductions in cerebral blood flow have been associated with migraine aura pathophysiology [18]. Additionally, research suggests that the depolarization of neurons and glial propagation in a wave-like manner across susceptible brain regions may be related to both migraine aura and RLS [19].

Previously reported prevalence rates of RLS in migraine patients have ranged from 8.7 % to 39.0 % [20, 21], with no significant differences between migraineurs with aura (MAs) and migraineurs without aura (MOs) [12, 22, 23]. Although research regarding the relationship between RLS and migraine frequency is limited, Cho et al. [24] have reported similarly increased prevalence rates of RLS among migraineurs who experience between one and ten attacks per month relative to those observed among migraineurs experiencing fewer than one attack per month. However, participants in the Cho et al. study were not divided into separate MA and MO groups.

Among migraine patients, RLS comorbidity has been observed to be associated with poorer sleep quality [12]. Additionally, the authors of the present study previously reported that higher migraine frequency is correlated with poorer sleep quality in both MA and MO patients [25]. The aim of the present study was to determine whether higher migraine frequency is correlated with increased prevalence of RLS and, if so, whether the relationship between migraine frequency and RLS prevalence differs between patients with MA and MO.

## Methods

### Patients

A cross-sectional controlled study was conducted with a cohort of 505 participants undergoing outpatient monitoring through the Department of Neurology at Tri-Service General Hospital (TSGH) between January of 2014 and December of 2015. The study protocol received approval from the Institutional Review Board of TSGH, and all participants provided informed written consent prior to enrollment.

Each participant completed a screening questionnaire and was subsequently interviewed by a board-certified neurologist and headache specialist (FCY) who made a diagnosis based on criteria defined in the International Classification of Headache Disorders, 3^rd^ edition (ICHD-III beta) [2]. Participants also completed the Migraine Disability Assessment Questionnaire (MIDAS), which is a 5-item questionnaire used to evaluate disability over the last 3 months [26]. The participants were then divided into the following four study groups: chronic headaches (≥15/month; *N* = 57), high-frequency headaches (9–14/month; *N* = 91), low-frequency headaches (1–8/month; *N* = 224), and migraine-free controls with no history of primary or secondary headache disorders (*N* = 133). Of the 372 participants with migraine, 111 (29.8 %) were patients with MA, and 261 were patients with MO.

### Patient assessments

#### Evaluation of RLS

RLS was diagnosed according to the following five essential diagnostic criteria outlined by the International RLS Study Group [27]: (1) urge to move the legs that is usually accompanied by unpleasant sensations in the legs; (2) urge to move or uncomfortable sensations that begin or worsen during periods of rest, including lying down or sitting; (3) urge to move or uncomfortable sensations that are relieved partially or totally by leg movement; (4) urge to move or uncomfortable sensations that worsen in the early evening or night compared with daytime, or occur only in the evening or at night; and (5) the inability of other medical or behavioral conditions (e.g., venous stasis, leg edema, myalgia, arthritis, positional discomfort, habitual foot tapping, leg cramps) to account for the aforementioned features.

None participants reported any history of physical, cognitive, or degenerative diseases of the central nervous system, or severe head injuries with loss of consciousness. All participants underwent detailed neurologic and electromyography examinations, as well as Doppler ultrasound examination by an experienced radiologist, in order to screen for any disturbances in the venous drainage of the lower extremities. No definitive abnormalities were identified for any of the patients. Patients exhibiting common potential causes for secondary RLS, including anemia, blood ferritin < 50 ng/ml, blood creatinine >1.5 mg/dl, and pregnancy, were also excluded. Diagnoses of idiopathic RLS were supported by physical, neurologic, electromyography, and radiologic examinations and laboratory data.

### Assessment of anxiety and depression

The Hospital Anxiety and Depression Scale (HADS) [28] is a 14-item scale used to assess symptoms of anxiety (7 items) and depression (7 items). Each item is scored from 0 to 3 (0 = not at all; 1 = sometimes; 2 = often; and 3 = all the time), yielding total possible scores within the range of 0–21 for anxiety and 0–21 for depression. We adopted the cut-off point of 8/21 for either anxiety or depression utilized by Bjelland et al [29].

### Quantification of sleep quality

The Pittsburgh Sleep Quality Index (PSQI) [30], which includes 19 self-rated items distributed among seven components, is designed to assess an individual’s quality of sleep during the past month; total PSQI scores (range: 0–21) ≥6 were considered indicative of sleep disturbance. Although we utilized the Chinese version of the MIDAS, HADS, and PSQI in the present study [31–33], each has been validated in a number of published studies [25, 34, 35].

### Data analysis

Data obtained for continuous covariates are presented as means ± standard deviations (SDs), while data obtained for categorical covariates are reported as numbers with proportions. Odds ratios (ORs) are reported with 95 % confidence intervals (CIs). The Cochran-Armitage chi-square test and univariate linear regression were used to investigate potential trends with regard to categorical and continuous covariates, respectively, in relation to the four group designations. The hypothesized relationship between migraine frequency and RLS prevalence was investigated using univariate analysis (the Cochran-Armitage chi-square test) as well as linear contrast in a multivariate logistic regression analysis adjusted for subject characteristics (variables listed in Table 1, except for aura and MIDAS). Finally, we employed both univariate and multivariable logistic regression analyses to determine which factors were associated with RLS prevalence. Data analyses were conducted with SPSS Version 22 (IBM SPSS, Armonk, NY: IBM Corp).

**Table 1:** Characteristics of the study population

Variable	No migraine controls (*N* = 133)	Episodic migraine groups			*p* value^§^
		1–8 days (*N* = 224)	9–14 days (*N* = 91)	≥15 days (*N* = 57)	
Aura	-	71 (31.7)	23 (25.3)	17 (29.8)	0.526
Female gender	89 (66.9)	150 (67.0)	64 (70.3)	39 (68.4)	0.666
Age (years)	35.4 ± 12.6	33.1 ± 10.2	33.5 ± 10.3	32.7 ± 12.4	0.165
Body mass index (kg/m^2^)	23.2 ± 4.0	22.0 ± 3.4	22.5 ± 4.0	22.4 ± 4.0	0.319
Education level (years)	14.6 ± 2.5	14.6 ± 2.6	14.5 ± 2.5	14.2 ± 2.9	0.315
Smoking status					0.001
Never	130 (97.7)	171 (76.3)	71 (78.0)	46 (80.7)	
Current or Former	3 (2.3)	53 (23.7)	20 (22.0)	11 (19.3)	
Alcohol consumption					0.098
Never	106 (79.7)	133 (59.4)	62 (68.1)	38 (66.7)	
Current or Former	27 (20.3)	91 (40.6)	29 (31.9)	19 (33.3)	
Coffee consumption					0.005
Never	53 (39.8)	52 (23.2)	24 (26.4)	10 (17.5)	
Less than once a month	39 (29.3)	43 (19.2)	23 (25.3)	21 (36.8)	
At least 1 day a week	41 (30.8)	129 (57.6)	44 (48.4)	26 (45.6)	
MIDAS	-	24.5 ± 19.3	37.6 ± 25.5	68.8 ± 58.1	<0.001
HADS–anxiety	6.0 ± 3.6	7.7 ± 3.9	8.3 ± 4.1	8.9 ± 4.6	<0.001
HADS–depression	4.6 ± 3.0	5.3 ± 3.9	6.6 ± 3.9	7.2 ± 4.6	<0.001
PSQI total score	7.4 ± 3.0	8.4 ± 3.5	10.0 ± 3.5	9.8 ± 4.4	<0.001

^§^Linear trend Cochran–Armitage chi-square test for categorical variables; linear contrast of univariate linear regression for continuous variables

## Results

### Participant characteristics

The demographic and clinical characteristics of participants in each study group are summarized in Table 1. Overall, less than a third of the patients experienced auras (111/372; 29.8 %). No trends in the distributions of gender, age, body mass index, education level, or alcohol consumption across the study groups were detected.

Linear trend analyses (unadjusted) revealed that more frequent migraine headaches correlated with higher MIDAS, HADS anxiety, and HADS depression scores, as well as with total PSQI score. Participants in the control group were less likely to be smokers and consumed coffee less often than participants in the migraine groups (Table 1).

### Effect of migraine frequency on RLS prevalence

**Fig. 1:**
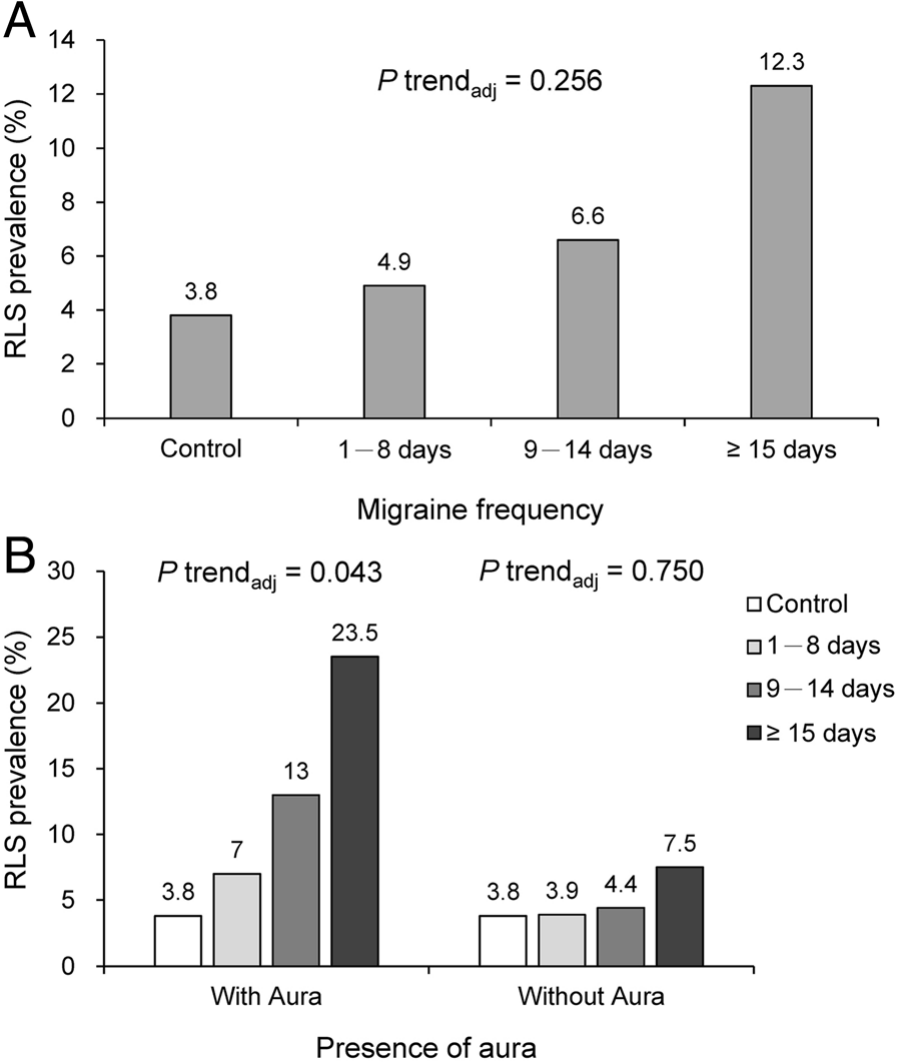
**a** RLS prevalence across the study groups. **b** RLS prevalence stratified by aura or no aura across the study groups.

The results of our analyses of the relationship between headache frequency group and RLS are reported in Table 2. Briefly, our univariate analysis revealed an effect of migraine frequency on total RLS prevalence (whole cohort), though the effect was no longer significant after adjusting for baseline characteristics. When we stratified the participants in the migraine groups into subgroups according to the presence of aura (i.e., MA and MO), we observed a robust significant relationship between migraine frequency and RLS prevalence in MA subgroups, but not in MO subgroups (Table 2). The total RLS prevalence rates for the headache frequency-delineated study groups as well as for the MA and MO subgroups are reported in Fig. 1a and 1b, respectively.

**Table 2:** Comparison of RLS prevalence across study groups

Population/RLS	No migraine controls (*N* = 133)	Episodic migraine groups			*p* value^†^	*p* value^§^
		1–8 days (*N* = 224)	9–14 days (*N* = 91)	≥15 days (*N* = 57)		
Whole cohort (*N* = 505)					0.026	0.256
Non-RLS	128 (96.2)	213 (95.1)	85 (93.4)	50 (87.7)		
RLS	5 (3.8)	11 (4.9)	6 (6.6)	7 (12.3)		
With aura (*N* = 244)					0.002	0.043
Non-RLS	128 (96.2)	66 (93.0)	20 (87.0)	13 (76.5)		
RLS	5 (3.8)	5 (7.0)	3 (13.0)	4 (23.5)		
Without aura (*N* = 394)					0.385	0.750
Non-RLS	128 (96.2)	147 (96.1)	65 (95.6)	37 (92.5)		
RLS	5 (3.8)	6 (3.9)	3 (4.4)	3 (7.5)		

^†^Linear trend analysis by Cochran–Armitage chi-square test (unadjusted analysis);

^§^Linear contrast in the multivariable logistic regression with adjustment of gender, age, body mass index, education level, smoking status, alcohol consumption, coffee consumption, HADS anxiety, HADS depression, and PSQI score

### Factors associated with RLS prevalence

The results of univariate and multivariate analyses aimed at elucidating those factors associated with RLS prevalence are reported in Table 3. The univariate analyses revealed that participants in the chronic migraine frequency group (≥15 days/month) were more likely to experience RLS than control participants. Higher HADS anxiety scores and total PSQI scores were also associated with higher RLS prevalence; both associations remained significant after adjusting for baseline characteristics (Table 3).

**Table 3:** Factors associating with RLS prevalence

Variable	Univariate analysis		Multivariable analysis	
	OR (95 % CI)	*p* value	OR (95 % CI)	*p* value
Migraine frequency				
Control (reference)	-	-		
1–8 days	1.32 (0.45–3.89)	0.612	1.03 (0.29–3.71)	0.963
9–14 days	1.81 (0.54–6.11)	0.341	1.21 (0.30–4.88)	0.791
≥ 15 days	3.58 (1.09–11.82)	0.036	2.34 (0.56–9.83)	0.246
Female gender	0.94 (0.43–2.05)	0.874	0.87 (0.35–2.18)	0.763
Age (years)	1.01 (0.98–1.04)	0.547	1.02 (0.98–1.06)	0.374
Body mass index (kg/m^2^)	1.02 (0.93–1.13)	0.663	1.00 (0.90–1.11)	0.946
Education level (years)	0.96 (0.84–1.10)	0.568	1.02 (0.88–1.19)	0.762
Current or former smoker	1.23 (0.49–3.10)	0.663	0.98 (0.33–2.96)	0.973
Current or former alcohol	1.86 (0.89–3.92)	0.100	1.55 (0.63–3.84)	0.340
Coffee consumption				
Never (reference)	-	-		
< once a month	0.71 (0.24–2.04)	0.518	0.59 (0.17–2.03)	0.402
≥ 1 day a week	0.96 (0.41–2.25)	0.923	0.97 (0.36–2.61)	0.947
HADS-anxiety	1.20 (1.09–1.31)	<0.001	1.18 (1.03–1.36)	0.019
HADS-depression	1.08 (0.99–1.18)	0.083	0.89 (0.78–1.02)	0.094
PSQI total score	1.26 (1.13–1.39)	<0.001	1.17 (1.02–1.33)	0.023

## Discussion

In the present study, we observed that increased RLS prevalence was associated with migraine frequency (based on number of headache days per month), utilizing an age- and gender-matched control group for the analysis. RLS prevalence correlated strongly with migraine frequency group in patients with MA after adjusting for well-known confounding variables (i.e., age, gender, body mass index, education level, smoking status, alcohol consumption, coffee consumption, MIDAS score, HADS anxiety score, HADS depression score, and total PSQI score). RLS prevalence was also associated with HADS anxiety score and PSQI total score.

In a recent nationwide, population-based cohort study conducted in Taiwan, we detected an increased risk of RLS in patients with migraine when compared with non-migraineurs, regardless of comorbidities or migraine subtype [36]. Further, Ferreira et al. [37] observed that RLS prevalence was higher in patients with migraine than in controls in a Brazilian cohort, though they did not analyze whether RLS prevalence differed with respect to presence of auras or headache attack frequency, for which the sample sizes were likely too small to provide sufficient power. More recently, a large population-based study that collected data from approximately 2300 patients has indicated that RLS is more prevalent and more severe in patients with migraine, and is also associated with poorer sleep quality. However, conditions mimicking RLS could not be excluded, which may have influenced the results [38, 39]. The results of the present study, consistent with those of previous studies [7, 8, 36–39], indicate that migraineurs have a higher prevalence of RLS than controls. Further, we observed particularly high RLS prevalence for patients with MA as well as an association between RLS prevalence and higher frequency of headaches.

These findings contrast with those of previous studies that have reported similar RLS prevalence rates between patients with MA (8.3–33.3 %) and MO (0.9–32.2 %) [12, 22, 23]. Also contrary to the findings of our study, Onofrio et al. reported that the RLS prevalence rate in patients with MA was similar to that of non-migraineur controls in Italy and that patients with MA and RLS seemed to have fewer attacks per month than patients with MA who do not have RLS, though the sample size (MA group *N* = 63) was relatively small [40, 41]. The aforementioned result discrepancies across studies may be related, at least in part, to differences in the populations examined. Indeed, the annual RLS incidence rate among non-migraineur controls (3.42/10,000 person-y) in our previous nationwide Taiwanese study was lower than that reported in a German cohort study (9–22/10,000 person-y) [36]. Moreover, in a systemic review, Schürks and colleagues observed lower RLS prevalence rates among Asians (1.8–5.6 %) than non-Asians (5.6–12.0 %) [9]. Therefore, the findings of the present study should be interpreted cautiously in recognition of potential differences in population ethnicities, as well as differences in methodology and clinical setting.

The reported associations between smoking/caffeine consumption and higher migraine frequency support the notion that both smoking and caffeine consumption may be significant triggers of migraine attacks [42]. Additionally, the observed linear trends between HADS scores and higher migraine frequency in the present study align with the claim that depression (63.8 %) and anxiety (60.4 %) are the most common comorbid conditions of migraine [43]. Furthermore, the observed association between migraine frequency and sleep disturbance (i.e., PSQI score) in the present study is consistent with the Vgontzas et al. finding that 48.8 % of patients with migraine experience some form of sleep disturbance, even after controlling for anxiety (lifetime and current) and the presence of other mood disorders [44].

Multivariate regression analyses indicated that anxiety (HADS anxiety score) and sleep disturbance (PSQI total score) were each an independent predictor of RLS prevalence (Table 3), with the latter result corroborating our previous finding that RLS is an independent predictor of poor sleep quality (PSQI total score ≥ 6) [25]. Therefore, it seems highly likely that RLS and poor sleep quality act to exacerbate each other.

Though Sevim et al. [45] observed a correlation between RLS severity and the severity of anxiety/depression symptoms, positing that disorders of anxiety and depression may be related to RLS symptoms, the results of the present study suggest a relationship between RLS and anxiety, but not depression. However, the present study utilized the HADS to assess symptoms of anxiety and depression, whereas Sevim and colleagues conducted diagnostic interviews. Further research is certainly warranted regarding RLS and comorbid anxiety and depression disorders.

Several lines of evidence suggest that the clinical features and underlying pathophysiology of both RLS and migraine may be related. Iron deposition in the brain has been reported in migraineurs, and repeated attacks have associated with increased levels of iron [46]. Conversely, iron deficiency has been implicated in the pathophysiology of RLS [11]. Further, dopamine-mediated premonitory symptoms [14] have been more commonly reported in patients with migraine and comorbid RLS than in patients with migraine and no RLS [15], and dopamine receptor antagonism can abort migraine attacks [47]. Meanwhile, central dysfunction of dopaminergic hypothalamo-spinal (A11) neurons has been strongly implicated in the pathophysiology of RLS, and the dopaminergic hypothesis has been supported by observation of beneficial effects of dopamine receptor agonist therapy on RLS symptoms [13]. Finally, a genetic analysis study by Fuh et al. [48] observed that presence of the Meis homeobox 1 gene was associated with increased risk of RLS in migraineurs and in patients with MA specifically.

The results of the present study have significant clinical implications. RLS, in and of itself, may impair sleep quality, which could play a direct role in the development of chronic migraine disorders. Furthermore, given that chronic migraine can not only be highly disabling and exert a significant negative impact on quality of life but also increase the relative socioeconomic burden associated with patient care [49], alleviation of RLS in migraineurs may improve quality of life and potentially prevent the development of chronic migraine in patients with episodic headaches [8]. Hence, the present study reinforces the need to identify potential cases of RLS in patients with migraine—particularly in patients with MA who have high-frequency attacks—in order to provide the most appropriate and effective treatments.

The strengths of the present study are its well-controlled design, demographically homogenous groups, analysis of MA versus MO subgroups, and robust statistical analysis. However, the study also had some limitations. First, the cross-sectional design restricts the causal relationship between migraine and RLS. Second, RLS prevalence (in Table 3) was established based simply on IRLSSG diagnostic criteria without consideration of severity. Third, sleep quality was evaluated using a self-reported index rather than an objective assessment. The mean PSQI score for our controls (7.4 ± 3.0; poor sleepers 75.4 %, data not shown) was higher than that reported by Seidel et al. [50] but similar to prior data from Taiwanese patients (6.1 ± 2.2; poor sleepers, 60.8 %) [51]. Finally, the relatively small numbers of participants in the chronic frequency group and MA patient subgroups may have reduced the potential to observe additional associations. Indeed, the relatively small cohort size in our study limited our ability to examine more differentiated subgroups, considering the interaction or influence by other migraine comorbidities, such as cardiovascular disorders, psychiatric conditions, obesity, epilepsy, fibromyalgia, and asthma [3–6, 52]. Therefore, future studies involving larger cohort sizes with more differentiated subgroups aid researchers in understanding the associations among various aspects and comorbidities of migraine.

## Conclusions

In conclusion, the results of the present study revealed a significant association between RLS prevalence and higher migraine frequency in patients with MA. The results further demonstrated that higher HADS anxiety scores and total PSQI scores were associated with higher RLS prevalence. Further, interventions targeted toward preventing the evolution of episodic disorders into chronic migraine conditions might reduce the tendency for comorbid RLS, particularly in patients with MA. Future studies should investigate those factors that relate RLS and migraine and aim to clarify the relationship between RLS severity and anxiety/depression.

## Abbreviations

HADS: hospital anxiety and depression subscales
IRLSSG: International RLS Study Group
MA: migraine with aura
MIDAS: migraine disability assessment questionnaire
MO: migraine without aura
PSQI: Pittsburgh Sleep Quality Index
RLS: restless legs syndrome
TSGH: Tri-Service General Hospital
